# An Investigation of a Cluster of Parapoxvirus Cases in Missouri, Feb–May 2006: Epidemiologic, Clinical and Molecular Aspects

**DOI:** 10.3390/ani3010142

**Published:** 2013-02-28

**Authors:** Edith R. Lederman, Min Tao, Mary G. Reynolds, Yu Li, Hui Zhao, Scott K. Smith, Lisa Sitler, Dana L. Haberling, Whitni Davidson, Christina Hutson, Ginny Emerson, David Schnurr, Russell Regnery, Bao-Ping Zhu, Howard Pue, Inger K. Damon

**Affiliations:** 1 Poxvirus and Rabies Branch, Division of Viral and Rickettsial Diseases, Centers for Disease Control and Prevention, Atlanta, GA 30333, USA; E-Mails: nzr6@cdc.gov (M.G.R.); lay4@cdc.gov (Y.L.); cgz9@cdc.gov (H.Z.); sks5@cdc.gov (S.K.S.); wfd6@cdc.gov (W.D.); zuu6@cdc.gov (C.H.); dtt4@cdc.gov (G.E.); russellregnery@mac.com (R.R.); iad7@cdc.gov (I.K.D.); 2 Epidemic Intelligence Service, Office of Workforce and Career Development, Centers for Disease Control and Prevention, Atlanta, GA 30333, USA; E-Mail: tao_minmin@yahoo.com; 3 Lincoln County Health Department, Troy, MO 63379, USA; E-Mail: sitlel@lchdmo.org; 4 Office of the Director, Division of Viral and Rickettsial Diseases, Centers for Disease Control and Prevention, Atlanta, GA 30333, USA; E-Mail: fnj2@cdc.gov; 5 Viral and Rickettsial Disease Laboratory, California Department of Public Health, Richmond, CA 94804, USA; E-Mail: david.schnurr@cdph.ca.gov; 6 Missouri Department of Health and Senior Services, Jefferson City, MO 65102, USA; E-Mails: baopingzhu@yahoo.com (B.-P.Z.); howard.pue@dhss.mo.gov (H.P.)

**Keywords:** parapoxvirus, orf virus, pseudocowpox virus, transmission, diagnostics, occupational exposure

## Abstract

**Simple Summary:**

In the spring of 2006, four human cases of parapoxvirus infections in Missouri residents were reported to the Centers for Disease Control and Prevention (CDC). We conducted surveys of herders and veterinarians, performed animal and environmental sampling and obtained sera from potential case-patients. We determined that, in general, infected persons may seek advice from veterinarians rather than physicians, thereby giving physicians less clinical experience. The initial perception of increased incidence in Missouri was likely due to reporting bias due to misdiagnosis and increased awareness due to recent publications. Basic personal protective measures are not being routinely utilized. Asymptomatic parapoxvirus infections in livestock may be common and warrants further investigation.

**Abstract:**

In the spring of 2006, four human cases of parapoxvirus infections in Missouri residents were reported to the Centers for Disease Control and Prevention (CDC), two of which were initially diagnosed as cutaneous anthrax. This investigation was conducted to determine the level of recognition of zoonotic parapoxvirus infections and prevention measures, the degree to which veterinarians may be consulted on human infections and what forces were behind this perceived increase in reported infections. Interviews were conducted and clinical and environmental sampling was performed. Swab and scab specimens were analyzed by real-time polymerase chain reaction (PCR), whereas serum specimens were evaluated for parapoxvirus antibodies. Three case patients were found to have fed ill juvenile animals without using gloves. Forty-six percent of veterinarians reported having been consulted regarding suspected human orf infections. Orf virus DNA was detected from five of 25 asymptomatic sheep. Analysis of extracellular envelope gene sequences indicated that sheep and goat isolates clustered in a species-preferential fashion. Parapoxvirus infections are common in Missouri ruminants and their handlers. Infected persons often do not seek medical care; some may seek advice from veterinarians rather than physicians. The initial perception of increased incidence in Missouri may have arisen from a reporting artifact stemming from heightened concern about anthrax. Asymptomatic parapoxvirus infections in livestock may be common and further investigation warranted.

## 1. Introduction

Parapoxviruses of small ruminants cause vesiculoulcerative disease of both keratinized skin and mucosal surfaces [1,2]. These viral agents include Orf virus, pseudocowpoxvirus, and bovine papular stomatitis virus; they are found worldwide, including the United States [2,3,4]. Humans with normal immune systems who have contact with infected animals or fomites develop self-resolving cutaneous ulcers, usually on the hands. Occasionally infections are of clinical significance in humans with normal immune systems, primarily due to bacterial superinfection [5]. A person with significant T cell dysfunction may develop atypical lesions (e.g., “giant” orf) and/or have protracted courses of illness [6,7,8,9]. As the disease is usually self-limited, infected humans often do not seek medical attention, especially if they are familiar with the disorder (e.g., reside in a farming community) [10]; as a result, the true disease burden among humans is unknown [11] and therapeutic options have not been well characterized. 

From February through May 2006, four human cases of parapoxvirus infections in Missouri residents were reported to the Centers for Disease Control and Prevention (CDC). Two were initially diagnosed as cutaneous anthrax, resulting in expenditure of scarce public health resources. One child with an active infection was barred from school because of communicability concerns. A public health investigation was initiated in part to ensure that, if a common source existed, it was identified prior to the start of summer fairs during which animals are commonly displayed for the public.

The objectives of this investigation were: (1) to determine whether the initial cluster of cases represented a regional increase in parapoxvirus infections, (2) to determine whether there was a common source for these infections (e.g., sale barn or use of the orf vaccine) and ongoing risk of transmission to humans (*i.e.*, infected animals), (3) to ensure additional information was made available to help primary care providers differentiate between parapoxvirus infections and cutaneous anthrax.

## 2. Materials and Methods

### 2.1. Case Investigation

All four cases and family members were interviewed using a standard questionnaire to collect basic demographic information, information pertaining to exposure to domestic ruminant animals (e.g., sheep, goats, dairy cattle), movement of animals on and off the farm and introduction of new animals, daily farm activities, number of years of animal exposure, glove use during animal care, and observations of parapoxvirus infections in their animals. In addition, individuals with a history of parapoxvirus infection (including the four primary cases) were questioned regarding the symptoms associated with their illness, health care seeking behavior, and treatments received. Samples were collected with dry swabs from both symptomatic domestic ruminants and bystander animals, and their pens. 

### 2.2. Herder Survey and Investigation

Farms neighboring case farms were selected on a referral basis (*i.e.*, referred by case farm owner) to serve as points of comparison. Owners, family members and employees were interviewed using a standard collection tool similar in nature to the one described above. This survey was supplemented with photographic aids of classic presentations of parapoxvirus infections in both humans and animals. If interviewees themselves had evidence of recent parapoxvirus infection (*i.e.*, active or resolving lesions) they were offered free testing to include swab of lesion (if lesion was active) or a blood draw for parapoxvirus serology (if lesion was resolving). In addition, such individuals were questioned regarding their symptoms, health care seeking behavior, and treatments received. Whenever possible, domestic ruminant animals were visually examined and swabs of their oral mucosa and the animal environment (e.g., pens, bedding, discarded wool and artificial milk nipples, *etc.*) were obtained. We utilized SAS V9.1 (Cary, NC, USA) for all statistical analyses. Odds ratios (ORs) with 95% confidence intervals (CIs) were calculated using logistic regression analysis.

### 2.3. Veterinarian Survey

A self-administered survey was mailed to veterinarians from the three counties in which human parapoxvirus infections had been confirmed and one adjacent county (Lincoln), as well as to state/federal veterinarians from Missouri Department of Agriculture and USDA (assigned to Missouri). This brief survey requested information regarding (1) frequency of consultation for parapoxvirus infections among animals, (2) frequency of consultation for parapoxvirus infections among humans (*i.e.*, herders), (3) knowledge of the use of the orf vaccine among local herders.

### 2.4. County/State Fair Investigation

In order to obtain a broader sample of Missouri livestock handlers, animal exhibitors of target species (goat, sheep, cattle) at 3 county fairs and the 2006 Missouri state fair were asked to respond to the ‘herder survey’ (see above). In addition, sheep from exhibits at the State Fair were examined by one investigator (H.P.) and oral swabs were obtained. The owners of selected sheep were queried regarding previous orf in their flock in the previous year. 

### 2.5. Laboratory Investigation

Swab and scab specimens were hydrated with 300 µL of phosphate buffered saline and centrifuged for 2 minutes in a swab extraction tube system (S.E.T.S., Roche Diagnostics Corporation, Indianapolis, IN, USA) per the manufacturer’s guidelines. DNA was extracted from 100 µL of elutant with a standard tissue protocol for the BioRobot EZ1 (Qiagen^®^) and the product was analyzed by real-time polymerase chain reaction (PCR) with parapoxvirus generic and orf specific assays. 

### 2.6. Parapoxvirus-Generic and Orf-Specific Real Time-Polymerase Chain Reaction Assay

The primer/probe sequences were selected from the multiple DNA sequences alignment of DNA polymerase gene (GenBank AY386263, NC_005336, NC_005337) with Primer Express (version 1.5; Applied Biosystems). These included:

(1) Parapoxvirus-generic assay:
  Parapox-generic forward primer (5'-GTA_CCG_CGC_CAT_GTC_CA-3')
  Parapox-generic reverse primer (5'-CCT_TCG_GCA_AGG_ACG_ACG-3')
  Parapox-genericprobe (5'-CC_AGC_GAG_TAG_TTC_GCG_TAC_ATG_TCC-3')
(2) Orf-specific assay:
  Orf forward primer (5'-GCT_CGC_TGG_CCA_CCA_G-3')
  Orf reverse primer (5'-CGG_GCC_CAT_GTC_CGT-3')
  Orf probe (5'-CT_CTA_TCT_CCG_AGC_GCT_GGC_GC-3')

Primers and probe were synthesized in the Biotechnology Core Facility (CDC, Atlanta, GA, USA), utilizing standard phosphoramidite chemistry. Primers and probe were synthesized in the Biotechnology Core Facility (CDC, Atlanta, GA, USA), utilizing standard phosphoramidite chemistry. The detection probe contained a 5' reporter molecule (FAM) and a 3' aminomodifier (both from Glen Research, Sterling, VA, USA). A 3' Blackhole quencher molecule (Molecular Probes, Eugene, OR, USA) was conjugated to the 3' amino group after synthesis.

Real time-PCR assay conditions were optimized according to standard protocols (protocol 04304449, Applied Biosystems, Foster City, CA, USA) by adjusting primer and probe concentrations, and thermal cycling temperatures/duration. Each reaction (30 µL) contained 2× Taqman universal PCR Master Mix (Applied Biosystems, Foster City, CA, USA), 25 µmol/L of each primer and probe, and 1 µL of template DNA. Thermal cycling conditions for the ABI PRISM 7900 (Applied Biosystems, Foster City, CA, USA): one cycle of 95 °C for 6 min; followed by 45 cycles of 95 °C for 5 s, and 60 °C for 25 s. PCR amplification is based on fluorescent emission after annealing/elongation (60 °C).

### 2.7. Parapoxvirus Serologic Assay

Serum specimens were evaluated by the following immunologic technique. Antibody to orf virus was detected by an indirect immunofluorescence assay [12] using pseudocowpox virus as the antigen. For IgG detection, serial two fold dilutions of sera, starting at 1:8 were added to pseudocowpox virus infected acetone fixed cell spots and incubated at 37 °C for 30 min. Slides were then washed in phosphate-buffered saline (PBS) and anti-human IgG (Panbio, Columbia, MD, USA) added and allowed to incubate for 30 min at 37 °C. The slides were then washed with PBS, mounted with cover slips in polyvinyl alcohol (PVA) and read with a fluorescence microscope. Results were reported as the reciprocal of the highest dilution at which specific fluorescence was observed. Titers of 1:8 or greater were considered positive. For IgM detection, serial two fold dilutions of MarSorb™ G (MarDx Diagnostics, Inc., Carlsbad, CA, USA) treated sera starting at 1:10 were tested with a primary incubation of 45 min at 37 °C, followed by a 30 min incubation with anti-human IgM conjugate (Panbio, Columbia, MD, USA) at 37 °C. Slides were washed, mounted and read using a fluorescence microscope. Titers of 1:10 or greater were reported as positive.

### 2.8. Sequencing and Phylogenetic Analysis

PCR amplified specimens were cleaned and concentrated according to standard protocols (short protocol, DNA Clean & Concentrator Kit, Zymo Research, Orange County, CA, USA) in order to remove unincorporated primers and dNTPs. The specimens were then sequenced according to standard protocols (BigDye Terminator v3.1 Cycle Sequencing Kit, applied Biosystems, Foster City, CA, USA). Each reaction (10 μL) contained 4 μL Terminator Ready Reaction Mix, 3 μL sterile dH_2_0 (to adjust total volume to 10 μL ), 1 μL of each sequencing primer (concentration 1.6 μM), and 2 μL of template DNA. Thermal cycling conditions: an initial denaturation at 96 °C for 1 min, followed by 96 °C for 10 s, 50 °C for 5 s, 60 °C for 4 min for 30 cycles followed by holding at 4 °C. 

The samples were then purified according to standard protocols (DyeEx 2.0 Spin Protocol for Dye-Terminator Removal, Qiagen, Valencia, CA, USA). 

DNA sequencing primers targeting a virion core protein gene (OV_SA00_027, GenBank NC_005336) and a virion morphogenesis gene (OV_SA00 108, GenBank NC_005336) were synthesized in the Biotechnology Core Facility (CDC, Atlanta, GA, USA), utilizing standard phosphoramidite chemistry:

Primers orf_027 for: 5' CGT CTA GAG CAT GCC CTC GTA and orf_027 rev: 5' CGA TCC TCG AGT CGC TTC TG generated a 440 bp DNA fragment and primers orf_108 for (AAG GCG AAC TCG TGC TTG A) and orf_108 rev (CCT CAT CAT TCT CGA CGA TAT G) generated a 870 bp DNA fragment.

The sequences of a virion core protein gene and a virion morphogenesis gene from clinical sample ORFV 031, 036, and 044 have been deposited in GenBank under accession no. FJ442820 and FJ460507, FJ460505 and FJ460508, FJ460506 and FJ460509, respectively. The sequences were concatenated and aligned with BioEdit [13] and Clustal alignment programs [14]. Phylogenetic analysis was performed using the Bayesian analysis software package (v1.7, http://beast.bio.ed.ac.uk), BEAST, BEAUti, and Tracer [15]. The analyses run MCMC chain length of 10,000,000 with a GTR + Gamma (category 4) + Invariant sites nucleotide rate substitution model, strict molecular settings and sampling of every 1,000 states. 

## 3. Results

### 3.1. Case Investigation

Characteristics of the four confirmed parapoxvirus cases reported to CDC by Missouri Department of Health and Senior Services are represented in Table 1. Three of four individuals who developed these infections were directly involved in the manual feeding of ill juvenile animals; cases did not use gloves while handling the ill animals. Two of the three operations (farms) were closed (did not obtain new animals in the past year). None of the case farms reported using the orf vaccine. We did not identify any additional recent cases (in the preceding year) among family members or employees. 

**Table 1:** Summary of four cases of human parapoxvirus infection reported to Centers for Disease Control and Prevention (CDC) by Missouri Department of Health and Human Services, February–May 2006.

Case	Sex	Age	Animal source	Animal overtly ill?	Risk factor/ animal contact	Virus identified	Closed operation? *	Used orf vaccine?	Glove use?
1	F	34	dairy calf;	yes	tube feeding	pseudocowpox virus	no	n/a ^†^	no
			family farm						
2 ^‡^	M	41	sheep;	yes	bottle feeding	orf virus ^§^	yes	no	no
			family farm						
3 ^‡^	M	10	sheep;	yes	bottle feeding	orf virus ^§^	yes	no	no
			family farm						
4	F	18	sheep;	no	shearing	orf virus	yes	no	no
			family farm						

^*^ closed operation: no new animals brought onto the operation in the previous year; ^†^ not applicable; ^‡^ father and son; ^§^ virus cultured in OAT3 cells (ATCC CRL-6546™).

### 3.2. Herder Survey and Investigation

**Figure 1:**
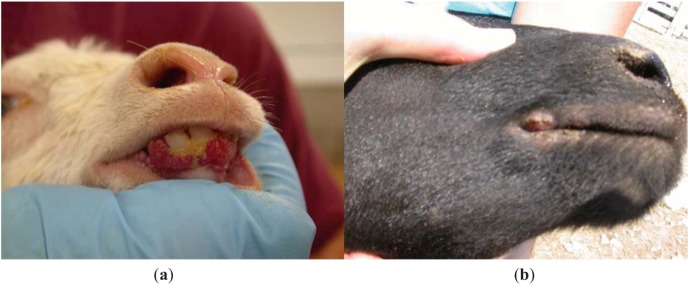
Orf clinical manifestations in a typical kid (**a**) *vs.* a lamb (**b**), Missouri, 2006.

Five additional farms in proximity to three of the four cases were evaluated with employee questionnaires and animal assessment/sampling; the fourth case (orf virus) was excluded because of lack of proximal farms and limitations in time for the investigation. Three of these five farms had overtly infected animals (sheep and goats). The kids examined were younger than the infected lambs (3–5 weeks old *vs.* 12–16 weeks old) and had more exuberant lesions, especially on their gingiva (Figure 1). Two additional cases of employees with orf lesions were identified during the evaluation of one neighboring dairy farm. As the lesions were healing and not amenable to confirmatory diagnosis using PCR, serology was performed to assess infection etiology as described above. Both employees had IgM and IgG titers to parapoxvirus ≥1:128. Both employees were young men from an urban area who had arrived several months before as part of a youth rehabilitation program; neither had prior farm animal exposure before being employed on this dairy. They both cared for kids (including bottle feeding) on a daily basis, several of which were actively infected at the time of our visit (infection status was confirmed by real-time PCR). 

In addition, questionnaires were administered to livestock owners at county fairs. A total of 97 questionnaires from individuals on community farms and attending Missouri county fairs were completed. Seventeen percent of respondents (n = 16) reported having at least one perceived parapoxvirus infection in the past. Thirty-five respondents reported the age at which they began farming and their current age, allowing us to compute the number of farm years for this subset (*i.e.*, potential for contact with parapoxvirus-infected livestock). These respondents had collectively 1,138 farm years and 3 infections yielding a rate of 26 infections/10,000 farm years. Table 2, Table 3 summarize the comparison of demographic, knowledge and risk factors evaluated for association with parapoxvirus infections. In the univariate analysis, age was not a significant risk factor, but male sex and having knowledge of parapoxvirus-associated conditions prior to the survey were significant risk factors;having an occupation listed as “other” (not a herder, student or homemaker) was protective. Only male sex (OR 5.1 [1.5–17.2]) remained an independent risk factor (*p* < 0.05) for parapoxvirus infection in the multivariate model (data not shown). Frequency and type of healthcare utilization as well as treatment for individuals with a history of parapoxvirus infections is described in Table 4. The majority of cases (13/21, 61.9%) sought healthcare during their presumptive infection, most (10/13, 76.9%) from a general practitioner. Among those evaluated by a health care provider, antibiotic prescription was common (11/13, 84.6%) and if a topical antiviral treatment were available in the future 19/22 (86.4%) of cases thought it would be worthwhile seeking healthcare to obtain a prescription. 

**Table 2:** Univariate analysis of demographics and knowledge for Missouri herders surveyed in 2006 with a self-reported history of parapoxvirus infections *vs.* those without such a history.

Characteristic	Ever Infected		No History of Infection			
	n (N = 22)	%	n (N = 81)	%	OR	95% CI
Sex						
Male	16	72.7	30	37.0	4.5^†^	1.6–12.8
Female	6	27.3	51	63.0	reference	
						
Occupation						
Herder (>50% livestock)	6	27.3	16	19.8	reference	
Others	16	72.7	65	80.3	1.5	0.5–4.5
						
Occupation						
Herder (any livestock)	11	50.0	23	28.4	reference	
Farm worker (non-owner)	2	9.1	2	2.5	2.1	0.36–16.9
Homemaker	2	9.1	5	6.2	0.8	0.4–5.0
Student	3	13.6	13	16.1	0.5	1.0–2.0
Others	4	18.2	38	46.9	0.2^*^	0.1–0.8
						
	**n (N = 21)**	**%**	**n (N = 80)**	**%**	**OR**	**95% CI**
Has heard of parapoxvirus infections before survey						
(missing 2)						
Yes	20	95.2	54	67.5	9.6 ^*^	1.2–75.7
No	1	4.8	26	32.5	reference	
						
	**n (N = 21)**	**%**	**n (N = 79)**	**%**	**OR**	**95% CI**
Know of others who have had parapoxvirus infection						
(missing 3)						
Yes	5	23.8	9	11.4	1.7	0.5–6.4
No	16	76.2	70	88.6	reference	
						

^*^ *p* < 0.05; ^†^ 0.001 < *p* < 0.01. OR = Odds Ratio, CI = Confidence Interval

**Table 3:** Univariate analysis of risk factors potentially associated with parapoxvirus infections: Missouri herders, 2006.

	Ever Infected		No History of Infection			
	n	%	n	%	OR	95% CI
Number of animals in contact with						
25–99	5/22	22.7	32/81	39.5	reference	
<25	3/22	13.6	17/81	21.0	1.1	0.2–5.3
>99	14/22	63.6	32/81	39.5	2.8	0.9–8.7
Smallpox vaccination						
(2 “unknown” status)						
Yes	12/20	60.0	56/69	81.2		
No	8/20	40.0	13/69	18.8	0.3	0.1–1.0
Daily contact						
Milking						
Yes	9/22	40.9	44/81	54.3	0.6	0.2–1.5
No	13/22	59.1	37/81	45.7		
Grooming						
Yes	19/22	13.6	65/81	80.3	1.6	0.4–5.9
No	3/22	86.4	16/81	19.8		
Bottle/Tube Feed						
Yes	19/22	86.4	66/81	81.5	1.4	0.4–5.5
No	3/22	13.6	15/81	18.5		
Birthing						
Yes	20/22	90.9	66/81	81.5	2.3	0.5–10.8
No	2/22	9.1	15/81	18.5		
Currently raise sheep						
Yes	15/22	68.2	55/81	67.9	1.0	0.4–2.8
No	7/22	31.8	26/81	32.1		
Currently raise goats						
Yes	3/22	13.6	9/81	11.1	1.3	0.3–5.1
No	19/22	86.4	72/81	88.9		
Currently raise cattle						
Yes	9/22	40.8	47/81	58.0	0.5	0.2–1.3
No	13/22	59.1	34/81	42.0		
Has seen infection in their						
livestock ever						
Yes	19/22	86.4	51/77	66.2	3.2	0.9–11.9
No	3/22	13.6	26/77	33.8		
Uses gloves ^*^						
Yes	17/22	77.3	31/80	38.8	2.2	0.7–6.4
No	5/22	22.7	49/80	61.3		
Has “facilitated” infection ^**^						
Yes	6/16	37.5	33/61	54.1	0.5	0.2–1.6
No	10/16	62.5	28/61	45.9		
Has used orf vaccine on sheep/goat						
Yes	8/19	42.1	22/61	36.1	1.3	0.5–3.7
No	11/19	57.9	39/61	63.9		
Has seen infection in animal within last year						
Yes	12/21	42.9	34/59	57.6	1.0	0.4–2.7
No	9/21	57.1	25/59	42.4		

^*^ uses gloves when handling sick animals and/or for routine care (*i.e.*, other than obstetrical/surgical procedures). ^**^ intentionally spread the infection from one animal to another to clear the infection more quickly from the herd/flock

**Table 4:** Clinical characteristics and healthcare seeking behaviors of humans with reported parapoxvirus infection: Missouri, 2006 (n = 22) ^*^.

Questions	Number (%) of Respondents Reporting “Yes” ^*^
Recall more than one parapoxvirus infection?	2/21 ^†^ (9.5)
Seek healthcare during the infection?	13/21 ^†^ (61.9)
If no, why?	
*Not severe enough*	–
*Already aware of diagnosis*	7/8 (87.5)
*No access to healthcare*	–
What type of physician(s) did you see? ^‡^	
*Urgent care*	2/13 (15.4)
*Family practice*	10/13 (76.9)
*Internal medicine*	–
*Sub-specialist (dermatology, infectious diseases)*	3/13 (23.1)
Repeat visits before diagnosis was made?	4/13(30.8)
Received antibiotics	11/13 (84.6)
If topical therapy were available, would this be worthwhile?	19/22 (86.4)

* includes the 6 confirmed cases in this investigation and 16 historical cases obtained during the herder interview; ^†^ survey(s) missing this data; ^‡^ case may have seen more than one physician type.

### 3.3. Veterinary Survey

Of the 52 veterinarians invited to participate, 33 surveys were returned. Five surveys were discarded (three were retired, one had no forwarding address, one was incomplete) leaving data from 28 respondents (54%) available for analysis. Sixty-four percent of respondents had been consulted for orf in sheep and goats at some point in their career; 32% reported having been consulted at least once a year. Only 29% percent of respondents reported ever having been consulted for pseudocowpox virus infection (in dairy cattle) during the course of their careers. Thirty-six percent surveyed were aware of the use of the live (non-attenuated) orf virus vaccine by local herders. Finally, 46% of veterinarians surveyed had been consulted for suspected human orf virus infection at some point; 18% reported being consulted at least once a year. Twelve percent of survey respondents had been consulted for human pseudocowpox virus infection at least once in their career.

**Figure 2:**
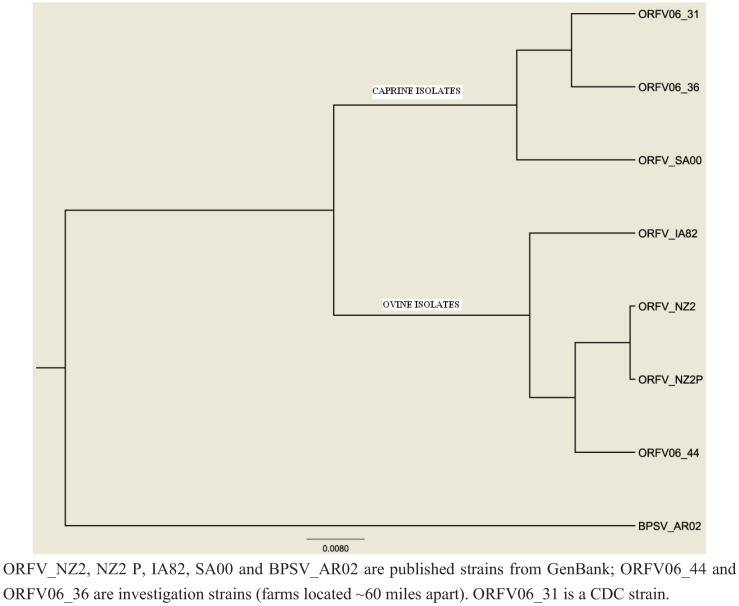
Phylogenetic tree of orf clinical samples inferred using a maximum likelihood approach.

### 3.4. Laboratory Investigation (Case, Herder and County/State Fair Investigation)

No overtly infected animals were found on any of the case farms. Swabs collected from previously infected, randomly selected animals and animal environments (pen walls, soil and bedding) were all negative for parapoxvirus DNA signatures (n = 12). Of the oral swabs obtained from domestic ruminants and/or environmental swabs from community farms 100% (7/7) sheep and goats with symptomatic disease as well as 21% (3/14) asymptomatic sheep and goats were positive for orf virus by real-time PCR. The only PCR-positive environmental sample was a swab of an artificial nipple used to feed kids at a commercial goat dairy. All sheep visually examined at the State Fair were free of overt signs of orf infection yet 20% (5/25) randomly sampled were positive for orf virus DNA via PCR, including two from a farm without report of any orf disease in the past year. 

Sufficient orf virus DNA was obtained from 2 animal samples (kid and lamb on community farms) to perform DNA sequencing. A virion core protein gene on the left end and an ATPase gene on the right end of the genome were partially sequenced, and the sequences were concatenated for the phylogenetic analyses to determine the relationships between the isolates collected and those of known strains of orf virus previously sequenced.

When gene sequences of the virus strains obtained from this investigation were compared to those from published sources and an additional virus archived at CDC, sheep and goat isolates tended to cluster in species-preferential fashion (Figure 2). This clustering occurred despite wide geographic distances and decades of time between the collection of isolates.

## 4. Discussion

Parapoxvirus infections of domestic ruminants (orf and pseudocowpox) are found worldwide. The results of our survey indicate that, not surprisingly, they are common among Missouri livestock (target species) and their handlers. Human orf cases were first reported in Missouri in 1983 [4]; coincident with the reports, a survey was conducted among Missouri sheep herders. At that time, 9% of herders reported having observed orf infections in their flocks. This value is somewhat lower than was observed in our investigation, where 17% of herders reported experience with the infection. This difference could be due to the smaller sample size of their survey (n = 47), or possibly to other differences in survey methods (e.g., of the use of visual aids in the current survey). Males in our survey were more likely to report a history of parapoxvirus infections; those individuals having “other” professions (less direct animal contact) were protected. 

The parapoxvirus infections on the three case farms that we investigated did not appear to be related. Since parapoxvirus infections are not reportable diseases in Missouri, we hypothesize that the perceived increase in incidence that triggered this investigation was in fact likely an artifact of reporting due to enhanced awareness (generated by a Morbidity and Mortality Weekly Report in January of 2006 [16]) or confusion with cutaneous anthrax, which occurred with two of the four cases and has occurred in similar settings abroad [17]. 

Bottle/tube feeding and general care of juvenile animals appears to be the primary route of transmission to humans in our investigation. This has been observed in other studies as well [3,10]. The “bummer” lamb (*i.e.*, a lamb orphaned by its ewe) poses some risk for parapoxvirus transmission to the caretaker because such animals are often bottle fed by hand and may have a weaker immune system and consequently may carry a higher viral burden. Providing care for these vulnerable young animals sometimes becomes the focus of 4H (a national youth organization sponsored by the Department of Agriculture) and other projects for schoolchildren, therefore, placing children at risk for avocational exposure [3,17]. The use of non-porous gloves while caring for an infected animal, especially during high risk behaviors (e.g., bottle feeding), is prudent [18,19]. Although we did not find any association between use of the orf vaccine and our six primary cases, infection from the vaccine in one Missouri herder has been reported [4] and veterinarians surveyed in the state were aware of its use. Therefore, history of orf vaccine use should be pursued when human cases are investigated. 

Because community and extension veterinarians have frequent interactions with the farming community, often serving as trusted advisors, they may be well-positioned to offer counseling regarding prevention of parapoxvirus transmission from animals to humans. Indeed, the results of our survey suggest that extension veterinarians see human parapoxvirus infections with some regularity. This may account for some underreporting of human infections (~50% of those surveyed in this report). This fact also decreases opportunities for practitioners of human medicine to become familiar with the infection, potentially contributing to misdiagnosis (*i.e.*, confusion with other zoonotic entities such as cutaneous anthrax) [20,21], and may lead to unnecessary antibiotic usage. In an effort to bridge this gap, information for clinicians regarding the distinguishing features of parapoxvirus infections, as opposed to cutaneous anthrax, was provided to Missouri Department of Health and Senior Services for distribution to clinicians and public health professionals. In addition, the Centers for Disease Control and Prevention and the Animal Plant Health Inspection Service have co-authored educational material regarding human infections with orf virus which is geared toward the farming community [22]. This material has been published by several industry circulars in an effort to reach the intended audience. Close collaboration between public health and animal health partners is essential in effectively preventing agriculturally-associated zoonoses.

The results of sampling of clinically asymptomatic sheep at the State Fair and community farms is intriguing as this activity provided evidence for asymptomatic infections. At least one case of an asymptomatic animal transmitting orf to a human during such interaction has been reported [10]. If asymptomatic infections indeed occur, then one could generate several hypotheses regarding virus transmission and local persistence. First, some animals may be chronically infected with orf virus and therefore represent reservoirs within the flock. Such animals may have partial immunity leading to subclinical infections without overt lesions, but sufficient virus production to contaminate common items (e.g., artificial nipples, as we found in our study). Alternatively, subclinical infections may wax and wane depending upon levels of stress. Although latent infections with poxviruses have not been definitively demonstrated, other DNA viruses, such as those belonging to *Herpesviridae* are prototypical examples of such persistent infections in other mammals. Vehicular transportation, crowding and exposure to new environments are all common stressors experienced by animals shown at a fair and could induce such rises in viral load if orf infection is capable of persisting in this manner. Likewise, a slaughterhouse, observed to be an excellent milieu for human transmission of orf virus [23] would further exacerbate stress. Therefore, longitudinal sampling of clinically asymptomatic flocks/herds is warranted to evaluate the potential for chronic, subclinical orf virus infections in sheep and goats. 

Phylogenetic analysis of isolates obtained during this investigation are limited by the small sample size but suggest that sheep and goat strains of orf virus could be inherently different and species preferential. Clinical distinctions were also observed; goats had more severe lesions and had overt disease at a younger age than sheep. However, these differences could also be attributed to host factors. More severe parapoxvirus-associated disease in goats has been well described, especially in particular breeds (Boer) [24,25]. Clinical severity in goats precludes the use of the commercially available live orf vaccine in goats [26]. Determining whether goat strains are inherently more pathogenic or whether goats are more susceptible to severe parapoxvirus-associated disease, may have implications for zoonotic transmission to human caretakers, especially given the rapid rise in the demand for goat meat in the U.S. The use of molecular diagnostics to distinguish human parapoxvirus infections is becoming increasingly common [27,28,29]. 

There are several limitations to our study. First, recall bias likely impacts reported estimates of previous parapoxvirus infections, especially given the potentially long period of time between the survey and the infection. In addition, we are basing our assumptions on the recall of laypersons. However, given the distinctive appearance, relatively long course of illness, the use of photographic aids during the interview and community’s familiarity with these infections, we feel that reasonably accurate data was obtained. Furthermore, studies of herders’ ability to diagnosis orf virus infections among their animals is fairly good (at least 80%) [30]. A second limitation stems from the fact that these data were collected from a convenience sample of Missouri herders and veterinarians and therefore information may not be broadly generalizable. Furthermore, the addition of cell culture evaluation to our molecular analysis of animal specimens would allow us to differentiate between true infection (viable virus) and the presence of DNA fragments (contamination from the environment *vs.* recent infection and clearance of the virus). However, some of the CT values in our “asymptomatic” animal specimens were reasonably strong (e.g., 36) and therefore difficult to dismiss as contamination. Furthermore, one of our cases (case 4), was infected during contact with an asymptomatic flock. Finally, the conclusions regarding species preference for particular strains of orf virus is intriguing but would be strengthened by comparing larger numbers of strains and possibly sequencing other variable genes for a similar comparison. 

## 5. Conclusion

In summary, parapoxvirus infections are common in Missouri livestock and their handlers. Humans infected with parapoxvirus often do not seek medical care; veterinarians are familiar with clinical presentations in both livestock and humans. Failure to present to human practitioners may lead to unfamiliarity and misdiagnosis, and ultimately, consumption of public health resources. Providing effective prevention messages targeted at the farming community (e.g., personal protective gear and good hand hygiene when handling an infected flock/herd) [21] and educating physicians on epidemiologic clues and key animal health questions are logical first measures in the prevention and identification of parapoxvirus infections in humans. This study supports the notion of asymptomatic infection in animals and possible predilection of certain strains for caprine *vs.* ovine hosts; the time course, degree of chronicity and impact on transmission to animals and humans alike have yet to be elucidated. 
